# Combining continuous glucose monitoring and insulin pumps to automatically tune the basal insulin infusion in diabetes therapy: a review

**DOI:** 10.1186/s12938-019-0658-x

**Published:** 2019-03-29

**Authors:** Martina Vettoretti, Andrea Facchinetti

**Affiliations:** Department of Information Engineering, University of Padova, Via G. Gradenigo 6/B, 35131 Padova, Italy

**Keywords:** Type 1 diabetes, Glucose sensors, Glucose prediction, Kalman filter, Hypoglycemia, Insulin pump

## Abstract

For individuals affected by Type 1 diabetes (T1D), a chronic disease in which the pancreas does not produce any insulin, maintaining the blood glucose (BG) concentration as much as possible within the safety range (70–180 mg/dl) allows avoiding short- and long-term complications. The tuning of exogenous insulin infusion can be difficult, especially because of the inter- and intra-day variability of physiological and behavioral factors. Continuous glucose monitoring (CGM) sensors, which monitor glucose concentration in the subcutaneous tissue almost continuously, allowed improving the detection of critical hypo- and hyper-glycemic episodes. Moreover, their integration with insulin pumps for continuous subcutaneous insulin infusion allowed developing algorithms that automatically tune insulin dosing based on CGM measurements in order to mitigate the incidence of critical episodes. In this work, we aim at reviewing the literature on methods for CGM-based automatic attenuation or suspension of basal insulin with a focus on algorithms, their implementation in commercial devices and clinical evidence of their effectiveness and safety.

## Background

Type 1 diabetes (T1D) is a chronic disease in which pancreas does not produce insulin, the hormone that stimulates transport of glucose from bloodstream to cells. To compensate the lack of endogenous insulin production, T1D subjects are treated with exogenous insulin, which is administered either by multiple daily injections or by an insulin pump. Insulin pumps are wearable medical devices that inject rapid-acting insulin in the subcutaneous tissue of the abdomen at almost continuous time, by a cannula connected to a disposable reservoir of insulin in the pump. A small basal dose is administered throughout the day to keep blood glucose (BG) concentration within the target range (70–180 mg/dl) in absence of external perturbations. Larger doses, called boluses, are injected before meals to cover carbohydrates intake or between meals to treat too high BG concentrations. Use of insulin pump was demonstrated to improve glycemic control in terms of glycated haemoglobin (HbA1c) values compared to multiple daily injections [1].

Insulin doses must be finely tuned in order to avoid dangerous complications. Insulin under dosing can drive to hyperglycemia (BG > 180 mg/dl) and eventually life-threatening short-term complications, such as diabetes ketoacidosis, or long-term complications such as cardiovascular diseases, kidney disease, retinopathy, neurological damage and diabetic foot. On the other hand, an over dosing of insulin can cause hypoglycemia (BG < 70 mg/dl) that, if severe and not promptly treated, can be catastrophic, causing loss of consciousness, seizures and even death. As a consequence, T1D subjects need to monitor their BG concentration during the day and adjust their insulin therapy accordingly.

Traditional home glucose monitoring consists in using self-monitoring of blood glucose (SMBG) devices that measure glucose concentration in small drops of capillary blood the patients collect 3–4 times per day by fingerstick [2]. More recently, continuous glucose monitoring (CGM) sensors have been introduced which can measure almost-continuously (e.g. every 5 min) glucose concentration in the subcutaneous tissue [3, 4]. Most of CGM sensors in the market are based on a needle electrochemical sensor which is implanted in the subcutaneous tissue of the abdomen or the arm. The sensor is connected to a transmitter that transfers in real-time the glucose readings to a portable receiver, which displays current glucose concentration and trend and produces audible alerts when glucose concentration exceeds user-defined hyper/hypoglycemia thresholds. Several studies demonstrated that CGM can improve diabetes therapy compared to SMBG, allowing to reduce both hyperglycemia and hypoglycemia frequency [5]. Since 2015, some CGM devices also received the regulatory approval for therapeutic use, i.e. CGM measurements can be used for making therapeutic decisions, like insulin dosing [4, 6, 7].

Although the use of insulin pump and CGM as standalone devices was demonstrated to be beneficial for glycemic control, the major potential benefits can be obtained by connecting the two devices in an integrated system. Indeed, even for the most motivated patients, the manual control of glycemia is a difficult task and requires subjects to interrupt their daily activities for making treatment decisions several times during the day. Manual glucose control is even more challenging during the night as subjects often do not wake up to CGM alarms [8]. Approaches to automatically tune insulin dosing based on CGM measurements have been an intense area of research in the last 10 years [9, 10]. As first step towards the development of a fully closed-loop system, also called artificial pancreas, academic research groups and companies investigated algorithms to automatically attenuate or suspend basal insulin infusion based on CGM measurements, e.g. when CGM detects hypoglycemia or CGM trend reveals a risk for imminent hypoglycemia [11, 12].

In this paper, we review the literature on methods for CGM-based automatic attenuation or suspension of basal insulin with a focus on algorithms, their implementation in commercial devices and clinical evidence of their effectiveness and safety.

## Automatic attenuation/suspension of basal insulin: definitions and classification

In the next sections, we will detail the algorithms and methods developed so far by academic groups and companies. Before that, in this section, we introduce some key concepts and definitions needed to highlight the differences between algorithms.

In general, the methods proposed for the automatic attenuation/suspension of basal insulin infusion can be divided into two categories: (i) those in which the attenuation/suspension is determined by the detection of hypoglycemia, and (ii) those in which the modulation of the basal insulin infusion pattern is triggered by the prediction of incoming hypoglycemia.

**Fig. 1:**
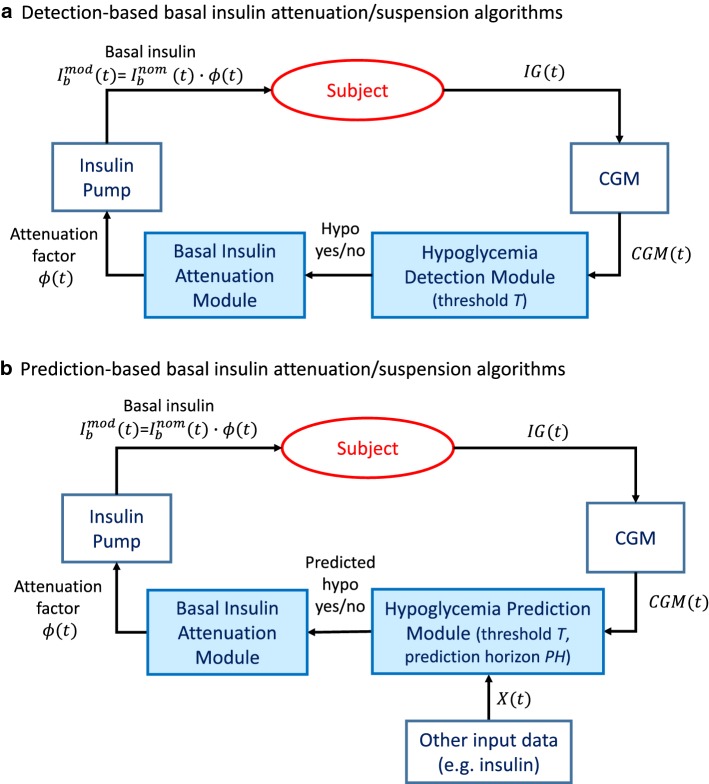
Schematic representation of basal insulin suspension/attenuation algorithms. **a** Schema of algorithms based on detection of hypoglycemia. Measurements of subject’s interstitial glucose (IG) concentration are real-time collected by a CGM sensor. When CGM measurements go below a threshold *T*, the *hypoglycemia detection module* detects hypoglycemia. Then, the *basal insulin attenuation module* calculates the attenuation factor $\phi (t)$, which is 0 if no hypoglycemia is detected and $0 <\phi (t)\le 1$ if a hypoglycemia is detected. Finally, the nominal basal insulin delivery rate, ${I_b}^{nom}(t)$, is multiplied for $\phi (t)$ to obtain the final modulated basal insulin delivery rate, ${I_b}^{mod}(t)$, which is given in output by the insulin pump. **b** Schema of algorithms based on prediction of hypoglycemia. In these algorithms, CGM measurements are used, optionally together with other input data (e.g. insulin), to predict in real-time the occurrence of hypoglycemic events *PH* min in advance (*hypoglycemia prediction module*). Such hypoglycemia prediction is then used to calculate a basal insulin attenuation factor, $\phi (t)$, as in detection-based methods

In *detection-based attenuation methods* (Fig. 1a), CGM measurements are real-time collected and when CGM data fall below a pre-defined threshold for hypoglycemia detection *T* (e.g. 70 mg/dl) a basal insulin attenuation factor $0\le \phi \le 1$ is calculated and multiplied for the nominal basal insulin injection rate (${I_b}^{nom}$) to reduce basal insulin delivery (${I_b}^{mod}$). When a stopping criterion is met, e.g. CGM readings return above the threshold *T*, the attenuation factor $\phi$ is set to 1 and the nominal basal insulin injection is restored. One limitation of detection-based algorithms is that the attenuation of basal insulin at the time of hypoglycemia detection can only reduce the duration of the hypoglycemic event, but cannot prevent the event from happening, especially because of the intrinsic delay of subcutaneous insulin absorption. For example, Swan et al. reported that, after a subcutaneous bolus of insulin, the peak of plasma insulin occurred 60 min after the bolus time [13]. Therefore, even if basal insulin is suspended when hypoglycemia is detected, there is still some previously-injected insulin active in the plasma causing a lowering in BG concentration.

To overcome this limitation, *prediction-based attenuation methods* can be used. Such strategies allow switching on/off the attenuation of basal insulin on the basis of the predicted CGM value, rather than of the current CGM reading (Fig. 1b). Specifically, in prediction-based methods, CGM measurements and optionally other input data, e.g. insulin doses, are used to forecast future glucose concentration. If a hypoglycemia is predicted to occur in the near future, i.e. predicted glucose concentration falls below a threshold *T*, then a basal insulin attenuation factor $0\le \phi \le 1$ is calculated and multiplied for the nominal basal insulin injection rate to reduce basal insulin delivery, thus possibly avoiding the predicted hypoglycemic event. When glucose is predicted to return in the safe range, the nominal basal insulin injection is restored to its nominal value ($\phi =1$). Besides the threshold *T*, another key parameter to tune the performance of prediction-based algorithms is the prediction horizon (*PH*), i.e. how much time in advance the hypoglycemic event would be predicted. Ideally, a longer *PH* would be desirable in order to anticipate the attenuation of basal insulin and thus compensate the delay of insulin action. However, in practice a too long *PH* is not convenient, because it increases the rate of false positives (i.e. predicted hypoglycemic events that do not happen in reality), and thus the risk of rebounds in hyperglycemia.

Finally, methods are classified as suspension algorithms if, instead of attenuating the basal insulin delivery, they simply interrupt basal insulin delivery when hypoglycemia is detected (*detection-based suspension methods*) or predicted (*prediction-based suspension methods*). In suspension algorithms, the attenuation factor $\phi$ can only be 0 or 1.

## Methods developed in academia

In the last 10 years, several detection- and prediction-based algorithms for basal insulin attenuation/suspension have been proposed in the literature, with the aim of preventing or mitigating hypoglycemia. Here, we illustrate the approaches developed so far, distinguishing those applying a pump suspension from those applying a modulation of basal insulin delivery. The characteristics of algorithms developed in academia are summarized in Table 1.

**Table 1:** Summary of literature algorithms for basal insulin attenuation or suspension proposed by academic research groups

Reference paper	Type	Inputs	Prediction method	PH, min	T, mg/dl	Min–max suspension time, min	Assessment type
Buckingham et al. Diabetes Technol Ther, 2009 [14]	Prediction-based suspension	CGM	Simple linear regression in time, statistical models [15]	30	80	90–90	Inpatient [14]
Buckingham et al. Diabetes Care, 2010 [16]	Prediction-based suspension	CGM	Voting schema of 5 separate prediction algorithms [17]	35	80	30–90	Inpatient [16]
Hughes et al. J Diabetes Sci Technol, 2010 [22]	Detection-based attenuation	CGM	–	–	120	–	In silico [22]
Hughes et al. J Diabetes Sci Technol, 2010 [22]	Prediction-based attenuation	CGM, insulin	Kalman filter with metabolic state observer	15	120	–	In silico [22]
Patek et al. IEEE Trans Biomed Eng, 2012 [26]	Prediction-based attenuation	CGM, insulin	Simple linear regression in time	17	112.5	–	In silico [26]
Cameron et al. J Diabetes Sci Technol, 2012 [19]	Prediction-based suspension	CGM	Kalman filter [18]	70	80	0-120	Inpatient [19], outpatient [20]
Buckingham et al. Diabetes Technol Ther, 2013 [20]	Prediction-based suspension	CGM	Kalman filter [18]	30	80	0–120	Outpatient [20, 38, 39, 46]
Hughes et al. Comput Methods Programs Biomed, 2013 [25]	Prediction-based attenuation	CGM, insulin	Simple linear regression in glucose with parameters from historical data	30	120 or 140	–	In silico [25]
Stenerson et al. J Diabetes Sci Technol, 2014 [21]	Prediction-based suspension	CGM, accelerometer, heart rate	Kalman filter [18]	30	80	0–120	In silico [21]

### Algorithms for basal insulin suspension

The first attempt to produce an algorithm to suspend the basal insulin infusion rate based on CGM values is due to the research group of Dr. Bruce Buckingham. Indeed, in 2009 Buckingham and colleagues developed and tested a method to discontinue insulin pump injection when hypoglycemia is predicted from real-time CGM [14]. The algorithm used in this study consisted in suspending basal insulin delivery for 90 min when glucose was predicted to fall below 80 mg/dl in the next 30 min. In particular, two methods were proposed for glucose prediction: a simple linear prediction algorithm that predicts future glycemia by linear extrapolation of CGM data; and a method using multiple empirical statistical models to estimate probability of future hypoglycemia [15]. The algorithms were assessed in a daytime 7-h in clinic experiment in which hypoglycemia was induced by controlled increase of basal insulin infusion rate.

After the encouraging results obtained in the first pilot study (56% of hypoglycemic events were prevented), Buckingham et al. further developed their method and tested a second prediction-based suspension algorithm for prevention of nocturnal hypoglycemia in clinic [16]. In this second study, hypoglycemia was predicted by using 5 different prediction algorithms and basal insulin infusion was suspended if 2 or 3 out of 5 prediction algorithms predicted a glucose value below 80 mg/dl in the next 35 min [17]. The 5 prediction algorithms considered were: linear extrapolation, Kalman filtering [18], adaptive hybrid infinite impulse filter, statistical prediction [15] and numerical logical algorithm. Instead of using a fixed suspension time of 90 min as in their previous study, the authors proposed a new criterion for early restart of basal insulin in case (i) at least 30 min passed since suspension started, (ii) CGM rate of change is more than 0.5 mg/dl/min, (iii) CGM value is greater than 80 mg/dl. The early restart criterion allowed to reduce the mean peak glucose after pump suspension from 158 ± 50 mg/dl to 149 ± 32 mg/dl. Among the 5 prediction algorithms, the only algorithm that did not play a significant role in the generation of hypoglycemia alarms was the linear extrapolation, result that justifies the investigation of more complex prediction algorithms.

In their subsequent work, Buckingham and coauthors abandoned the idea of having multiple prediction algorithms in favor of using a single Kalman filter prediction algorithm with a *PH* of 70 min [19]. This choice was motivated on one hand by the need to reduce computational complexity, on the other hand by the necessity to further anticipate the insulin suspension in order to increase the proportion of nights with prevented hypoglycemia, which was about 75% in their previous study [16]. For this purpose, the criteria to restart the basal insulin were also updated. In work by Cameron et al. [19], basal insulin delivery was restarted if glucose was predicted to rise above 100 mg/dl in the next 70 min. Moreover, the algorithm allowed a maximum suspension time of 120 min every 150 min and 180 min per night. The new algorithm was tested in clinic in 16 subjects. Mean suspension time was 89 min, hypoglycemia was prevented in 11/15 subjects, but 13/15 subjects had a peak glucose > 180 mg/dl after basal suspension. Nevertheless, the authors stated their study was not designed to rigorously assess the rebound in hyperglycemia, because hypoglycemia was artificially induced by increasing basal insulin delivery, and no control subjects were studied.

In 2013, Buckingham et al. [20] conducted an outpatient study to test the safety and effectiveness of the algorithm proposed by Cameron et al. [19] under real-life conditions. The trial revealed that the proposed algorithm was able to reduce overnight hypoglycemia (occurring in 19% of intervention nights vs 26% of control nights), but at the cost of significantly increasing mean morning glycemia (158 ± 52 mg/dl in intervention nights vs 125 ± 53 mg/dl in control nights). The authors then decided to revise their algorithm to reduce the occurrence of pump suspension. Specifically, the *PH* was reduced to 30 min, pump suspension was not allowed if CGM value was > 230 mg/dl or a drop of $\ge$ 40 mg/dl was present in consecutive CGM readings (suspected sensor compression artifact), and insulin delivery was resumed as soon as CGM values started rising. With the revised algorithm, overnight hypoglycemia occurred in 16% of intervention nights vs 30% of control nights and a smaller increase in mean morning glucose concentration was observed (144 ± 48 mg/dl vs 133 ± 57 mg/dl).

The algorithm by Buckingham et al. [20] was extended in work by Stenerson et al. [21] to incorporate physical activity measurements derived from accelerometer and/or heart rate monitor to improve the algorithm’s performance in mitigating exercise-induced hypoglycemia. Specifically, the extended algorithm suspended basal insulin delivery when CGM reading was below 180 mg/dl and decreasing and one of these two conditions was satisfied: (i) > 0.1 vector magnitude units on the accelerometer; (ii) > 90 beats per minute on the heart rate monitor. The efficacy of the algorithm was first tested in a simulated experiment. Results suggested that adding accelerometer data allowed to improve the performance of the pump suspension algorithm, while further addition of heart rate monitor data did not significantly improve the performance of the system.

### Algorithms for basal insulin attenuation

Starting from 2010, the research group led by Prof. Boris Kovatchev at the University of Virginia has been working on a different approach, i.e. algorithms to attenuate, rather than suspend, the basal insulin delivery according to the risk of hypoglycemia. The two main approaches that they developed were called *Brakes* and *Power Brakes* [22].

**Fig. 2:**
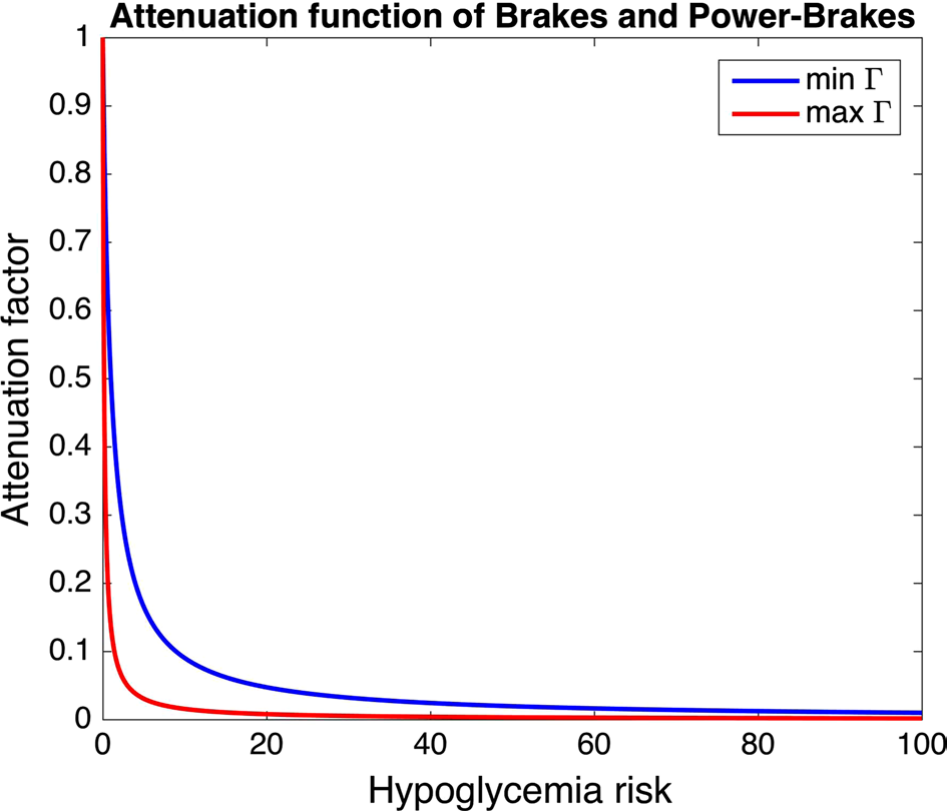
Attenuation function used in the *Brakes* and *Power Brakes* algorithms. Plot of the attenuation function used in the *Brakes* and *Power Brakes* algorithms (see Eq. 1) for two virtual subjects of the UVA/Padova T1D Simulator [58]. In particular, the function is plotted for the subject with maximum aggressiveness parameter (max $\Gamma$, red line) and the subject with minimum aggressiveness parameter (min $\Gamma$, blue line)

*Brakes* is a detection-based algorithm which attenuates basal insulin delivery when the 15-min unweighted moving average of CGM measurements, $\bar{y}$, goes below $T=120$ mg/dl with negative derivative. The attenuation factor $\phi$ is calculated according to the following formula:

1 $$\begin{aligned} \phi (R(\bar{y})) = \frac{1}{1+\Gamma \cdot R(\bar{y})} \end{aligned}$$

where $R(\bar{y})$ is the glucose risk function introduced by Kovatchev et al. [23] and $\Gamma$ is a patient-specific aggressiveness parameter derived from total daily insulin and correction factor. As visible in Fig. 2, where $\phi (R(\bar{y}))$ is plotted for two different values of $\Gamma$, the attenuation factor rapidly decreases as the hypoglycemia risk increases and this decrease is more rapid for higher $\Gamma$ values. Basal insulin delivery is restored as soon as the derivative of $\bar{y}$ becomes positive.

*Power Brakes* is a prediction-based algorithm that uses both CGM and insulin data to forecast glucose concentration in the next 15 min and attenuate basal insulin delivery accordingly. Specifically, glucose prediction is performed by the Kalman filter using as underlying model the metabolic state observer, i.e. a linearized and discretized version of the subcutaneous oral glucose minimal model [24] with population parameters. The inputs of the model are CGM measurements and insulin pump delivery rate. The basal insulin attenuation factor is calculated as in *Brakes* (Eq. 1 replacing $\bar{y}$ with $\hat{y}$, i.e. the 15-min ahead predicted glucose concentration returned by the Kalman filter). Basal insulin delivery is restored as soon as the derivative of $\hat{y}$ becomes positive.

The method of *Power Brakes* was further developed in work by Hughes et al. [25] by adding in input to the algorithm information extracted from historical CGM data collected in the previous 30 days. The goal was to let the algorithm anticipate routine behavioral events that can cause hypoglycemia, like exercise or consistent over-delivery of insulin. In the version of *Power Brakes* enhanced with historical CGM data, the Kalman filter and the metabolic state observer are used in real-time to filter the CGM signal and obtain a clean estimate of current glucose concentration. This is then projected in the next 30 min using a simple linear regression model with patient-specific parameters that depends on the time of the day (updated every 30 min) and have been estimated from historical CGM data collected in the previous 30 days. Historical data are also used to tune the threshold *T* for activation of basal insulin attenuation, which was set to either 120 mg/dl or 140 mg/dl depending on the probability of being in hypoglycemia in the next 60 min estimated from historical CGM data.

Another variation of the *Power Brakes* algorithm was proposed by Patek et al. [26] as safety supervision module (SSM) of a modular closed-loop system. In this variant, glucose prediction is performed by a simple linear regression in time with $PH=17$ min, intercept parameter equal to the unweighted average of CGM measurements collected in the last 10 min and slope parameter equal to the least-square linear fit of CGM samples in the last 30 min. Predicted glucose is then corrected to account for the effect of insulin-on-board (IOB), i.e. previously injected insulin that is still active in the body. This is done by subtracting to the predicted glucose concentration a term proportional to an estimate of current IOB, obtained using insulin action curves of different duration (4–8 h) in different glucose ranges. The glucose value obtained after IOB correction is used to calculate the risk of hypoglycemia using the same function of the original *Power Brakes* formulation, but with different parameters. Particularly, in the SSM variant, basal insulin attenuation is triggered only if CGM is lower than 112.5 mg/dl with decreasing trend. Finally, the attenuation factor is calculated as in Eq. 1, but without applying the aggressiveness parameter ($\Gamma =1$).

## Algorithms embedded in commercial devices

Since the availability of first CGM sensors, insulin pump companies understood the potentiality of exploiting CGM data in real-time to improve insulin infusion and, thus, glucose control.

**Fig. 3:**
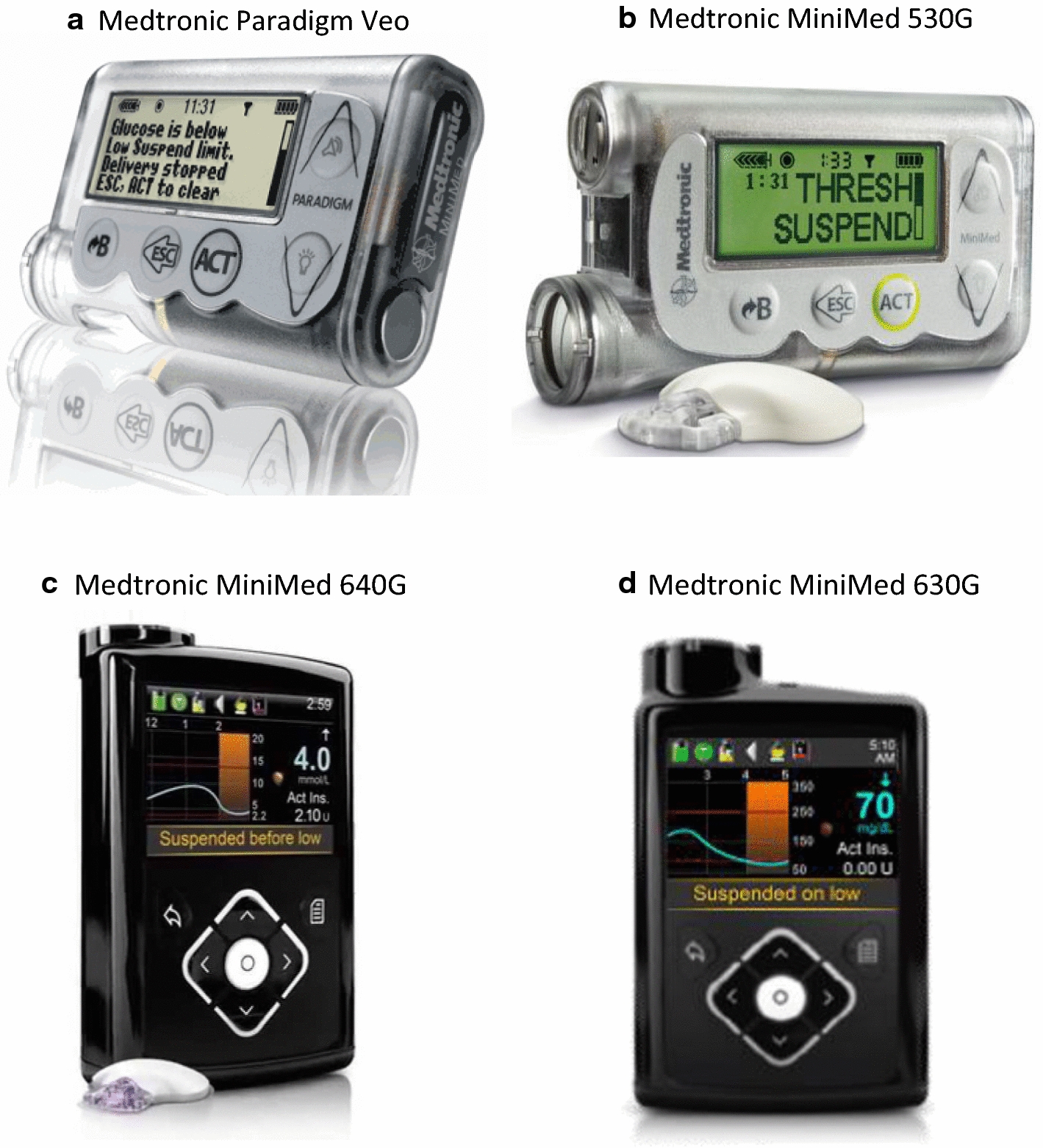
Commercial devices implementing basal insulin suspension algorithms. **a** Medtronic Paradigm Veo (taken from: http://www.desang.net/2011/02/medtronic-paradigm-veo). **b** Medtronic MiniMed 530G (taken from: http://www.medtronicdiabetes.com/loop-blog/introducing-the-minimed-530g-with-enlite). **c** Medtronic MiniMed 640G (taken from: http://www.medtronic-diabetes.co.uk/minimed-system/minimed-640g-system). **d** Medtronic MiniMed 630G (taken from: http://www.diabetesms.com/products/minimed-630g-insulin-pump)

Medtronic Inc. (Northbridge, CA) has been one of the most active companies working at the development of both detection- and prediction-based suspension algorithms. Indeed, Medtronic Inc. launched in 2006 the first system for diabetes management integrating CGM and insulin pump technologies in the same medical device (MiniMed Paradigm REAL-time system). Later on, Medtronic Inc. developed the Medtronic Paradigm Veo (Fig. 3a), i.e. the first device equipped with an automatic detection-based basal insulin suspension feature, which became commercially available outside the U.S. in 2009. The Paradigm Veo system implemented the low glucose suspend (LGS) feature that suspended basal insulin delivery for up to 2 h when the CGM value measured by the sensor embedded in the system fell below a low glucose threshold, which could be set by the user between 40 and 110 mg/dl. In particular, when the pre-defined threshold was reached, in absence of patient’s actions, a 6-h cycle automatically started, in which basal insulin was suspended for 2 h and then re-activated for the next 4 h regardless of CGM measurements. The fixed 4-h re-activation of insulin delivery was introduced to prevent the occurrence of diabetes ketoacidosis. Nevertheless, the patient could manually interrupt the LGS cycle at any time and resume normal basal insulin delivery [27]. In the U.S., the first device including automatic detection-based suspension of basal insulin, the MiniMed 530G system (Fig. 3b), entered the market several years later, in 2013. The system implemented the threshold suspend (TS) feature, which was identical to LGS, except for the range of settable low thresholds that was restricted to 60–90 mg/dl in this second device [28].

According to a retrospective study of Paradigm Veo data uploaded on Carelink Therapy Management Software for Diabetes (Carelink), the LGS feature was generally well accepted by the European users, who used the feature on 82% of the time, with the low threshold most commonly set between 50 and 60 mg/dl (data collected from January to July 2010) [27]. A similar analysis of MiniMed 530G data uploaded on Carelink showed that 70% of patients always used TS, 19% used it intermittently and the remaining 11% never used the feature (data collected from October 2013 to July 2014) [28]. Among MiniMed 530G users, the mean low threshold setting was 62.8 ± 5.8 mg/dl. However, even though the patients had the LGS/TS feature active on most of the time, actually, most of suspension events were manually interrupted by the users. Indeed, the retrospective analysis of Carelink data showed that about 50% of suspension events lasted 5 min or less and only 10% of events had complete 2-h duration [27, 28]. This suggests that a 2-h suspension may be too long for most of the events, thus users preferred to reactivate basal insulin earlier and/or make their own treatment.

A new prediction-based suspension algorithm, called “suspend before low” or Prediction Low Glucose Management (PLGM), was implemented in the SmartGuard feature of the MiniMed 640G (Fig. 3c), which became commercially available outside the U.S. in 2015. The system automatically suspends basal insulin delivery when glucose concentration is predicted to reach or fall below a preset glucose threshold in the next 30 min and automatically restarts basal insulin on recovery from hypoglycemia [29]. Specifically, basal insulin delivery is suspended if glucose concentration is below *T* + 70 mg/dl and predicted to drop below *T* + 20 mg/dl in the next 30 min, where *T* is a low threshold that the user can set between 50 and 90 mg/dl. The system automatically resumes basal insulin if glucose is $\ge T$ + 20 mg/dl, the 30-min ahead predicted glucose value is $\ge T$ + 40 mg/dl and basal insulin delivery has been suspended for at least 30 min. As in the LGS/TS feature, the user can manually restart basal insulin delivery at any time and the maximum duration of suspension events is 2 h, after which basal insulin delivery is resumed for 4 h regardless of CGM concentration. In the U.S., the SmartGuard feature became commercially available in 2016 as part of the MiniMed 630G system (Fig. 3d). Besides the new PLGM algorithm, the SmartGuard feature of both MiniMed 640G and MiniMed 630G implements the LGS/TS algorithm as well, which is also called “suspend on low”.

An analysis of MiniMed 640G real-world data (data uploaded on Carelink from January 2015 to January 2016), showed that “suspend before low” was used on 83% of user-days, “suspend on low” was used on 11% of user-days, while in the remaining 6% of user-days neither “suspend before low” or “suspend on low” were activated [30]. With the MiniMed 640G, the percentage of suspension events manually interrupted by the user decreased to 33.51%, 11% of events had maximum duration of 2 h, while in the remaining 55% of events basal insulin delivery was automatically resumed by the PLGM algorithm.

## Evidence of clinical effectiveness and safety

Many studies were performed to assess the effectiveness and the safety of basal insulin suspension algorithms both in clinic under controlled conditions [14, 16, 19, 31–35] and at home under real-life conditions [20, 29, 36–43]. The effectiveness is intended as the ability of the algorithm to reduce or prevent hypoglycemia and was generally assessed by metrics like number/rate of hypoglycemic events, time spent in hypoglycemia, percentage of values in hypoglycemia, minimum glucose value (or nadir) per suspension event and area under the curve (AUC) for hypoglycemic excursions. The safety is intended as the ability of the algorithm not to produce rebounds in hyperglycemia and adverse events, like diabetes ketoacidosis, that can result from an extended suspension of basal insulin delivery. Metrics commonly used to assess algorithm’s safety are mean glucose concentration, glycated hemoglobin (HbA1c), time spent in hyperglycemia, percentage of values in hyperglycemia, glucose values at restart of basal insulin, 2 h and 4 h after restart of basal insulin, morning glycemia and morning blood ketones.

The Medtronic LGS/TS algorithm was tested both in single-arm [36, 37] and randomized controlled clinical trials [31, 40, 41]. Evidences from these trials support the effectiveness of the algorithm in reducing hypoglycemia, at the price of a slight increase in hyperglycemia that, however, was not associated with an increase of HbA1c or the occurrence of ketoacidosis. For example, in the ASPIRE In-Home study [41], a randomized trial in which 247 subjects were randomly assigned to sensor-augmented pump therapy (SAP) or SAP + LGS for 3 months, AUC for nocturnal hypoglycemic events was reduced by 37.5% in the LGS group compared to the control group. Regarding the 2-h suspension events, 4 h after the event start, 26% of CGM values were above 200 mg/dl and the mean CGM value (168 ± 64.6 mg/dl) was pretty close to the hyperglycemic region. Nevertheless, no significant change in HbA1c was observed. In a 6-month randomized study comparing SAP + LGS therapy with pump therapy (no CGM) in patients with hypoglycemia unawareness, the rate of severe (coma and seizure) and moderate (requiring other person assistance) hypoglycemic events decreased from 129.6 to 28.4 events per 100 patient-months with no significant change in HbA1c [40]. In a partial analysis conducted in 24 patients [44], the investigators found a discrepancy between first morning CGM value, that was 144 ± 9 mg/dl, and first morning SMBG value, that was 185 ± 9 mg/dl, after overnight 2-h suspension events (n = 324). Moreover on 14 occasions, there were more than one 2-h suspension per night. In these cases, mean morning meter value was 239 ± 22 mg/dl and mean morning sensor value was 112 ± 18 mg/dl. The authors stated these events were caused by sensor’s calibration error, underlying the importance of relying on an accurate CGM sensor. These clinical trials also confirmed the evidence reported in Agrawal et al. [27, 28] that most of LGS/TS suspension events were manually interrupted by the user, with a percentage of events of duration less than 10 min ranging between 48% [44] and 66% [37].

An investigational Medtronic device with the PLGM feature was assessed by Abraham et al. in experiments of insulin-induced [33] and exercise-induced [34] hypoglycemia and under real-life conditions [43, 45], demonstrating the effectiveness of the algorithm in reducing hypoglycemia and hypotreatment requirements. The commercial MiniMed 640G system was assessed in a single-arm outpatient study [29], a single-arm inpatient study with nocturnal hypoglycemia induced by increased insulin delivery [35], and a randomized controlled trial conducted in children and adolescents [42]. In study by Choudhary et al., mean suspension event duration was 56.4 ± 9.6 min, mean of subsequent CGM nadir was 71.8 ± 5.2 mg/dl and CGM did not reach the preset low threshold in 83.1% of events [29]. Basal insulin delivery was manually restarted by the user in 26.7% of events, result of the improved strategy for basal insulin resumption in the PLGM feature. In in-clinic experiments, the 60% of hypoglycemic events (defined as two consecutive BG samples $\le$ 65 mg/dl) were prevented with PLGM [35]. In their randomized clinical trial comparing SAP with and without PLGM, Battelino et al. found that PLGM allowed a significant reduction of number of events below 65 mg/dl, at the price of an increase of time above 140 mg/dl, which was significantly longer in the PLGM group [42]. Nevertheless, time above 180 mg/dl and time above 250 mg/dl were comparable in the two groups. Moreover, no significant difference in the number of events below 50 mg/dl was observed, indicating that basal insulin suspension may not be sufficient to avoid episodes of profound hypoglycemia. The superiority of PLGM compared to LGS/TS was proved by Danne et al. in silico using the UVA/Padova T1D simulator [32]. Zhong et al. analysed the data of 851 users who transitioned from Paradigm Veo to MiniMed 640G and observed a statistically significant reduction of excursions below 70 mg/dl and above 300 mg/dl [30].

As far as noncommercial algorithms are concerned, the algorithm based on Kalman filter prediction developed by Buckingham et al. [20] was tested for reduction of overnight hypoglycemia in a large 42-night randomized controlled clinical trial involving 45 adolescents and adults, 45 children aged 11–14 and 37 children aged 4–10 [38, 39, 46]. The algorithm was running in a bedside laptop communicating with the Paradigm Veo pump and the Medtronic Enlite sensor. Results showed that the prediction-based suspension algorithm was effective in reducing hypoglycemia in all age groups. In adults and adolescents, median hypoglycemia AUC was reduced by 81% and the percentage of nights with at least one CGM value < 60 mg/dl was 21% in the intervention group versus 33% in the control group [38]. In children, median time below 70 mg/dl was about 50% lower in the intervention group. However, in subjects aged 11 or older, the percentage of nights followed by morning glycemia > 180 mg/dl was statistically significantly higher in the intervention groups compared to the control groups (42% vs 32% in 11–14 year-olds; 27% vs 21% in > 14 year-olds) [38, 39]. Despite the higher occurrence of morning hyperglycemia in patients using the suspension algorithm, no significant increase in morning blood ketones and no serious adverse event was observed [39, 46].

All the aforementioned clinical trials were performed with either detection-based or prediction-based suspension algorithms. The attenuation algorithms proposed by University of Virginia, *Brakes* and *Power Brakes*, were assessed in silico using the first release of the UVA/Padova T1D Simulator [47]. In Hughes et al. [22], two simulated scenarios were generated in which hypoglycemia was induced by increasing the basal insulin delivery rate and by an insulin bolus with missed meal, respectively. In the first scenario, both *Brakes* and *Power Brakes* were effective in reducing the number of subjects experiencing hypoglycemia (from 59% to 26% and 7%, respectively). In the second scenario, *Brakes* was able to reduce the duration of hypoglycemic events but did not succeed in preventing any of them. Better results were achieved by *Power Brakes*, which, taking into account previously injected insulin for glucose prediction, was able to prevent 32/90 hypoglycemic events. Hughes et al. also showed that combining *Power Brakes* with a hypoglycemic treatment advisor, which suggests 15 g of rescue carbohydrates when predicted glucose goes below 80 mg/dl, almost all the hypoglycemic events were prevented [22].

The variant of *Power Brakes* with historical CGM data as additional input was tested in silico in an ad-hoc scenario generated by the UVA/Padova T1D Simulator, in which a random increase in the patient’s basal rate was introduced to artificially simulate a circadian increase in the patient’s insulin sensitivity [25]. Results showed that the addition of historical CGM data allowed to modestly improve the performance of *Brakes* in preventing hypoglycemia, although an assessment of these techniques in either real clinical studies or more realistic in silico experiments is necessary to better understand the potential benefits of using historical data to enhance pump attenuation algorithms.

The SSM variant of *Power Brakes* was also assessed in silico, in a single-day experiment generated by the UVA/Padova T1D Simulator, in which patient’s insulin sensitivity and basal insulin rate were perturbed to generate a suboptimal treatment scenario [26]. Compared to the conventional therapy, the SSM was able to effectively reduce hypoglycemia (0% time in hypoglycemia), at the price of an upward shift of the glucose profiles. However, this shift was compensated when the module of the closed-loop system responsible for recommending both positive and negative basal insulin adjustments (Range Correction Module) was activated under the supervision of the SSM.

Although the encouraging results obtained in these short in silico experiments, to the best of our knowledge, *Brakes* and *Power Brakes* were never assessed as standalone algorithms in clinical trials, but only in closed-loop control trials, where they have been embedded in the SSM, making impossible to distinguish their effectiveness from that of the closed-loop controller. In addition, the effectiveness and safety of such algorithms have never been tested in more realistic long-duration in silico clinical trials when new and more reliable simulators were released [48, 49].

## Conclusion

The availability of insulin pumps communicating with CGM sensors has stimulated the development of algorithms for the automatic suspension or attenuation of basal insulin delivery when hypoglycemia is either detected or predicted from CGM readings. Some algorithms were also implemented in commercial integrated systems produced by Medtronic. The commercially available algorithms are all suspension algorithms, i.e. they can only turn on or off basal insulin delivery, and rely only on CGM measurements for the prediction of future hypoglycemia [27–29].

Literature studies have shown that the performance of glucose prediction algorithms can significantly improve when additional inputs are considered apart from CGM, like insulin, meals, and physical activity, all factors that strongly impact glycemic excursions [50–52]. Some literature studies on basal insulin attenuation algorithms have explored the possibility of enhancing glucose prediction by taking into account insulin data [22, 26] and historical CGM data [25]. However, such algorithms have been tested only in short in silico experiments and a more extensive evaluation is needed to understand the real benefits of using insulin and historical glucose data as additional inputs for glucose prediction. A first step in this direction could be the assessment of these algorithms using a more advanced simulation framework including a description of physiological, technological and behavioral variability, which allows the simulation of realistic multiple-day scenarios [48, 49]. In another literature study, physical activity data were used to enhance the performance of an algorithm for basal insulin suspension in mitigation of exercise-induced hypoglycemia [21]. However, in the proposed algorithm, physical activity could trigger basal insulin suspension independently on glucose prediction. A more robust approach would be to use physical activity data to enhance glucose prediction and then use the prediction derived from CGM, physical activity and possibly other input data to trigger basal insulin suspension.

Another interesting idea that has been proposed in the literature is the use of a smoothed attenuation function [22, 25, 26]. Further assessments are needed to understand the real benefits of using a smooth attenuation function instead of a threshold-based suspension strategy, like that implemented in commercial devices. Indeed, the plot of Fig. 2 shows that the attenuation function proposed by Hughes et al. [22] is very steep, thus the effect on glucose concentration of using such an attenuation function may actually be very similar to that obtained with a step function.

The assessment of basal insulin suspension algorithms in clinical trials showed that these methods are effective in reducing hypoglycemia outcomes, except the rate of events of profound hypoglycemia (e.g. below 50 mg/dl), whose incidence and duration did not change significantly with or without the use of pump suspension [22, 42]. The price is a slightly increase of time spent in the high glucose range (e.g. above 140 mg/dl) [38–40, 42], although no serious adverse event (e.g. diabetes ketoacidosis) associated with the use of suspension algorithms was observed.

As Hughes et al. shown in a simulated case study [22], the simple attenuation of basal insulin when hypoglycemia is predicted may not be sufficient to prevent episodes of profound hypoglycemia caused by large insulin boluses, unless this is combined with carbohydrate intake. Therefore, a possible effective solution for the prevention of profound hypoglycemia could be the combination of basal insulin attenuation algorithms with algorithms for the suggestion of preventive hypotreatments in presence of elevated hypoglycemia risk. Such preventive hypotreatment advisory systems could be also applied to mitigate hypoglycemia in patients on traditional multiple daily injections therapy.

To minimize the undesired rebound in the high glucose range following a suspension event, basal insulin attenuation algorithms can be coupled with algorithms allowing automatic basal insulin increase in response to high glucose concentration. This strategy was supported, for example, by Patek et al. that showed the performance of their SSM increased when this was coupled with the Range Correction Module allowing positive insulin corrections [26]. In recent work by Spaic et al., the prediction-based suspension algorithm developed by Buckingham et al. [39] was combined with an automatic insulin-dosing component, forming the Predictive Hyperglycemia and Hypoglycemia Minimization system for overnight control [53]. Two recent randomized clinical trials showed that the addition of the insulin-dosing component allowed the system to increase time in target and reduce mean morning glycemia, without deteriorating the performance in hypoglycemia mitigation [53, 54]. Systems like these that allow real-time automatic positive and negative adjustments of basal insulin delivery are examples of hybrid closed-loop systems, which have been object of intense research activity in the last 10 years and recently entered the market, with the Medtronic MiniMed 670G being the first commercial hybrid closed-loop system [55–57].

In conclusion, basal insulin attenuation algorithms are a promising technique for the mitigation of hypoglycemia in SAP therapy and represent the first step towards a fully closed-loop system. Although some of these algorithms have already been implemented in commercial devices, there are still many margins for improving their performance. Interesting research topics in this area include the enhancement of prediction algorithms by adopting multivariable approaches, the use of different smooth attenuation functions, and the combination of basal insulin suspension algorithms with preventive hypotreatment advisory systems and/or automatic insulin dosing algorithms.

## Abbreviations

T1D: Type 1 diabetes
BG: blood glucose
CGM: continuous glucose monitoring
HbA1c: glycated hemoglobin
SMBG: self-monitoring of blood glucose
PH: prediction horizon
SSM: safety supervision module
IOB: insulin-on-board
LGS: low glucose suspend
TS: threshold suspend
PLGM: Prediction Low Glucose Management
AUC: area under the curve
SAP: sensor-augmented pump
